# The Antarctic Circumpolar Current isolates and connects: Structured circumpolarity in the sea star *Glabraster antarctica*

**DOI:** 10.1002/ece3.4551

**Published:** 2018-10-12

**Authors:** Jenna M. Moore, Jose I. Carvajal, Greg W. Rouse, Nerida G. Wilson

**Affiliations:** ^1^ Florida Museum of Natural History University of Florida Gainesville Florida; ^2^ Scripps Institution of Oceanography UCSD La Jolla California; ^3^ Western Australian Museum Welshpool Western Australia Australia; ^4^ University of Western Australia Crawley Western Australia Australia

**Keywords:** Antarctica, cytochrome c oxidase subunit 1, Echinodermata, internal transcribed spacer region 2, phylogeography, Scotia Arc

## Abstract

**Aim:**

The Antarctic Circumpolar Current (ACC) connects benthic populations by transporting larvae around the continent, but also isolates faunas north and south of the Antarctic Convergence. We test circumpolar panmixia and dispersal across the Antarctic Convergence barrier in the benthic sea star *Glabraster antarctica*.

**Location:**

The Southern Ocean and south Atlantic Ocean, with comprehensive sampling including the Magellanic region, Scotia Arc, Antarctic Peninsula, Ross Sea, and East Antarctica.

**Methods:**

The cytochrome c oxidase subunit I (COI) gene (*n* = 285) and the internal transcribed spacer region 2 (ITS2; *n* = 33) were sequenced. We calculated haplotype networks for each genetic marker and estimated population connectivity and the geographic distribution of genetic structure using Φ_ST_ for COI data.

**Results:**

*Glabraster antarctica* is a single circum‐Antarctic species with instances of gene flow between distant locations. Despite the homogenizing potential of the ACC, population structure is high (Φ_ST_ = 0.5236), and some subpopulations are genetically isolated. Genetic breaks in the Magellanic region do not align with the Antarctic Convergence, in contrast with prior studies. Connectivity patterns in East Antarctic sites are not uniform, with some regional isolation and some surprising affinities to the distant Magellanic and Scotia Arc regions.

**Main conclusions:**

Despite gene flow over extraordinary distances, there is strong phylogeographic structuring and genetic barriers evident between geographically proximate regions (e.g., Shag Rocks and South Georgia). Circumpolar panmixia is rejected, although some subpopulations show a circumpolar distribution. Stepping‐stone dispersal occurs within the Scotia Arc but does not appear to facilitate connectivity across the Antarctic Convergence. The patterns of genetic connectivity in Antarctica are complex and should be considered in protected area planning for Antarctica.

## 1 INTRODUCTION

Population connectivity in benthic marine species depends on extrinsic environmental and oceanographic factors and biological features of behavior and development. Pelagic larval stages allow long‐range dispersal in benthic organisms; however, indirect estimates of dispersal ability such as planktonic larval duration (PLD) can be surprisingly unreliable in predicting the geographic distribution of benthic adults (Lester, Ruttenberg, Gaines, & Kinlan, 2007; Paulay & Meyer, 2006; Shanks, 2009). Dispersal ability can be modified behaviorally, for example, by rafting (Helmuth, Veit, & Holberton, 1994; Highsmith, 1985; Nikula, Fraser, Spencer, & Waters, 2010), which can result in differences between PLD‐predicted and realized dispersal. Genetic proxies, such as *F*_ST_, show consistent but only moderate correlation with PLD (Selkoe & Toonen, 2011; Weersing & Toonen, 2009), however, aggregate studies may underestimate this correlation (Dawson, 2014; Dawson, Hays, Grosberg, & Raimondi, 2014). Larval type and PLD are strongly correlated with local temperatures and productivity (Marshall, Krug, Kupriyanova, Byrne, & Emlet, 2012), and connectivity at high latitudes may be constrained by strong seasonality in reproduction and primary productivity. Organisms in the Southern Ocean have evolved reduced feeding in planktonic larval stages compared to species at lower latitudes (Marshall et al., 2012; Thorson, 1950), but the effect of this evolutionary tendency on dispersal remains unknown in many taxa.

Currents, water mass isolation, nutrient dynamics, and distance between suitable habitats are important in establishing species ranges. The Southern Ocean is an extreme environment in many of these respects, and drivers of dispersal and connectivity in the Antarctic fauna remain poorly understood. The Antarctic Circumpolar Current (ACC) is an unusually strong barrier to the north‐south exchange of organisms owing to its thermal and density‐driven isolation of water masses, as well as strong eastward flow. Estimates of the timing of ACC formation range from 20 to 41 Ma, corresponding with the opening of Drake Passage and the Tasman Seaway (Barker & Thomas, 2004; Ladant, Donnadieu, & Dumas, 2014; Lagabrielle, Goddéris, Donnadieu, Malavielle, & Suarez, 2009; Scher & Martin, 2006; Sijp et al., 2014). Intensification of the ACC at the Miocene‐Pliocene boundary is correlated with the timing of genetic separation of several benthic marine species pairs from the Magellanic region and the Antarctic continent, suggesting that water mass isolation generated by the ACC has had an important impact on Antarctic marine biotic isolation (Poulin, González‐Wevar, Díaz, Gérard, & Hüne, 2014).

Most of the Antarctic continental shelf is narrow and isostatically depressed, with a mean depth of about 200 m (http://www.gebco.net/), and is subject to high disturbance via seasonal ice scour and iceberg groundings (Gutt, 2001). During the last glacial maximum (LGM), grounded ice shelves are estimated to have covered much of the continental shelf, significantly decreasing available habitat for the shelf fauna (Anderson, Shipp, Lowe, Wellner, & Mosola, 2002; Huybrechts, 2002). However, some refugial habitat on the shelf and in deeper water must have persisted through glacial periods (Allcock & Strugnell, 2012; Anderson et al., 2002; Thatje, Hillenbrand, & Larter, 2005). Differential use of refugia would result in populations varying in signals of expansion. Glacial survival in deep sea refugia is supported by eurybathy in several groups of Antarctic invertebrates (Brey et al., 1996) and genetic signatures of long‐term population stability in many benthic species (Allcock & Strugnell, 2012).

In Antarctica, morphological similarity in widespread taxa suggests a circum‐Antarctic distribution in many species (Mackintosh, 1960). Recent molecular studies demonstrate cryptic speciation in many of these, repeatedly challenging this paradigm (Allcock et al., 2011; Brasier et al., 2016; Held, 2003; Held & Wägele, 2005; Krabbe, Leese, Mayer, Tollrian, & Held, 2009; Linse, Cope, Lörz, & Sands, 2007; Wilson, Hunter, Lockhart, & Halanych, 2007). Organisms with restricted dispersal may be more prone to cryptic divergence (Pearse, Mooi, Lockhart, & Brandt, 2009), with the most extreme Antarctic example known in the widespread complex *Doris kerguelenensis* (Bergh 1884), which represents at least 32 cryptic species (Wilson, Maschek, & Baker, 2013; Wilson, Schrodl, & Halanych, 2009). Truly circum‐Antarctic distributions have been corroborated by molecular data in very few taxa (Arango, Soler‐Membrives, & Miller, 2011; Dömel, Leese, & Convey, 2015; Hemery et al., 2012; Raupach et al., 2010; Strugnell, Watts, Smith, & Allcock, 2012).

Despite isolation between South America and the Antarctic Peninsula at the Antarctic Convergence, there remains a great deal of faunal overlap between these regions. The Scotia Arc is a chain of islands, seamounts and ridges spanning the Antarctic Convergence, providing areas of shallow shelf habitat in the Scotia Sea. These habitats may act as “stepping‐stones”, allowing dispersal across isolated water masses. Most recent studies on genetic connectivity in Antarctic marine invertebrates have generally focused sampling in the Scotia Arc region (e.g., Hoffman, Peck, Linse, & Clarke, 2011; Hunter & Halanych, 2010; Janosik, Mahon, & Halanych, 2011); however, there are few explicit tests of this stepping‐stone hypothesis (but see Wilson et al., 2007), and only recent studies include circumpolar sampling for comparison (Galaska, Sands, Santos, Mahon, & Halanych, 2017; Soler‐Membrives, Linse, Miller, & Arango, 2017).

**Figure 1:**
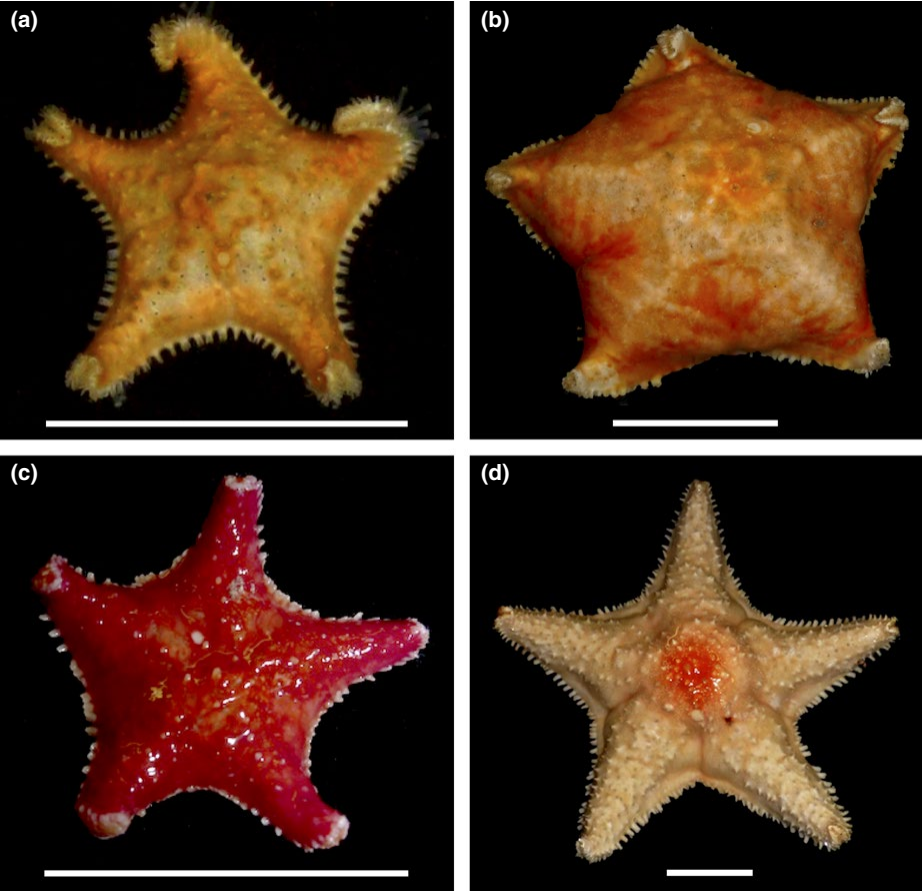
Morphological diversity in *Glabraster antarctica*. Specimens from (a) Bransfield Strait (Antarctic Peninsula), (b) South Georgia (Scotia Arc), (c) Burdwood Bank (Magellanic), (d) Shag Rocks (Scotia Arc). Scale bar is 35 mm

*Glabraster antarctica* (E. A. Smith, 1876; Figure 1) is a sea star commonly encountered on continental shelf and slope habitats in Antarctica, the subantarctic, and the Straits of Magellan, from the subtidal to 2,930 m (GBIF, 2014). A recent systematic revision of Poraniidae moved the species from *Porania* to *Glabraster,* and synonymized two geographically and morphologically defined subspecies: *Porania antarctica antarctica* E. A. Smith, 1876, and *P. antarctica magellanica* Studer, 1876 (Mah & Foltz, 2014). Three species, *Porania spiculata* and *Porania glaber*, both Sladen, 1889 and *Porania armata* Koehler, 1917 were already previously synonymized (Clark, 1993; Mah & Foltz, 2014) Our sampling covers the newly synonymized type localities and morphotypes, and thus provides an opportunity to evaluate the synonymy.

The pelagic brachiolaria larvae of *G. antarctica* have large yolk stores, but are facultatively planktotrophic (Bosch, 1989; Rivkin, Bosch, Pearse, & Lessard, 1986) and may disperse over large distances. This species’ putatively circumpolar distribution extending across the Antarctic Convergence, abundance, and high dispersal potential make it an excellent model system to investigate genetic connectivity in the Antarctic and subantarctic region on several geographic scales.

Here, we use evidence from two genetic markers to assess three hypotheses:

1. *Glabraster antarctica* is a single panmictic circum‐Antarctic species.
2. This connectivity is facilitated by the Antarctic Circumpolar Current.
3. Populations of *G. antarctica* maintain genetic connectivity across the Antarctic Convergence via “stepping‐stone” dispersal along the Scotia Arc.

## 2 MATERIALS & METHODS

### 2.1 Sample collection

**Figure 2:**
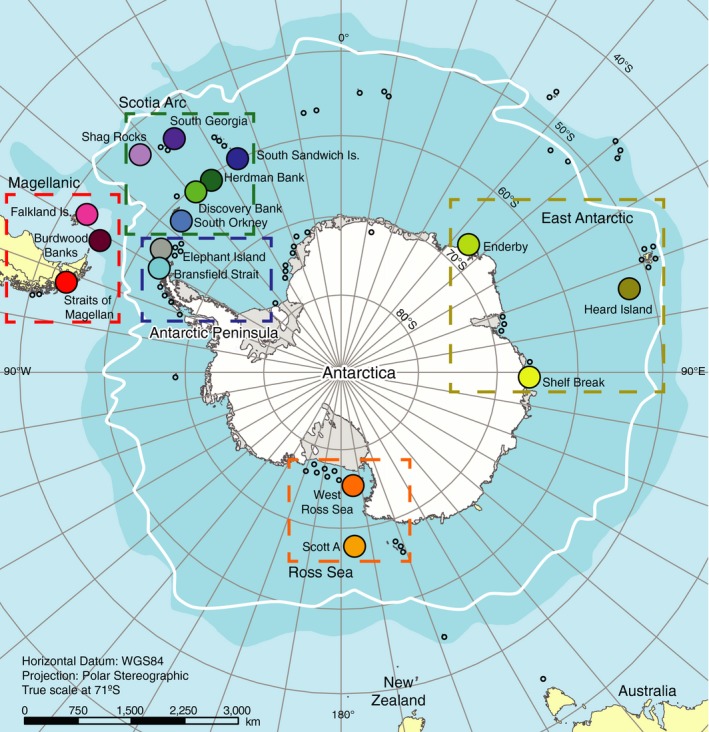
Map of sampling localities. Closed, colored circles indicate sites used in this study. Some encompass multiple sample sites. Open circles indicate summarized occurrence records of *Glabraster antarctica* obtained from the Global Biodiversity Information Facility Portal (GBIF). Dashed boxes indicate a priori geographic regions referenced in the text. Shaded blue area indicates average boundary of the Subantarctic Front, and white line indicates average location of Polar Front and the clockwise‐flowing Antarctic Circumpolar Current

*Glabraster antarctica* (*n* = 285) were sampled from 19 sites around the Antarctic continent (Figure 2, Appendix S1). Specimens from the Scotia Arc were collected from soft sediments by benthic Blake trawl during two expeditions in 2011 and 2013 aboard the RVIB *Nathaniel B. Palmer* (NBP11‐05 & NBP13‐03). Voucher specimens and field photographs were examined qualitatively for variation in abactinal spines for each site to assess the validity of subspecific names. Tissue samples preserved in 95% ethanol were kept cold until DNA extraction. Voucher specimens are housed at the Scripps Institution of Oceanography's Benthic Invertebrate Collection (SIO‐BIC) in La Jolla, California (Appendix S1). Additional samples were obtained from ethanol‐preserved collections at the National Institute of Water and Atmospheric Research (NIWA, New Zealand) and the Australian Antarctic Division (AAD).

### 2.2 Genetic data collection

Genomic DNA was extracted using a Qiagen DNeasy Blood & Tissue Kit according to the manufacturer's instructions and diluted 100–200‐fold for amplification. The cytochrome c oxidase subunit I mitochondrial gene (COI; *n* = 285) was amplified in PCR using the COIceF/COIceR primer set (Hoareau & Boisson, 2010), with an annealing temperature of 45°C. Considerable efforts to amplify nuclear markers across the sample were unsuccessful; these included ATPS5, ATPS7, EFInt4 introns (Foltz, Nguyen, Nguyen, & Kiger, 2007), ATPS5, GPI (Keever et al., 2009), and ANT, Cyc A, and Calmodulin (Audzijonyte & Vrijenhoek, 2010). The internal transcribed spacer region 2 (ITS2; *n* = 33) was PCR‐amplified with difficulty using an annealing temperature of 42°C using the forward primer JW‐5.8SL (Waters & Roy, 2003) and a new reverse primer designed using Primer3 (Untergrasser et al., 2012): JC‐3814R (5′‐ TCCTCCGCTTAGTGATATGCT‐3′). Being a small and fairly unrepresentative sample, ITS2 data were only used to ascertain whether high levels of diversity were also present in the nuclear genome, to corroborate mitochondrial diversity. Successful PCR products were purified using ExoSAP‐IT and outsourced for Sanger sequencing to Eurofins MWG Operon (Louisville, KY, USA). Sequences were trimmed and checked for errors in Geneious Pro 6.1.7 (Biomatters, Ltd.), then aligned using MUSCLE (Edgar, 2004).

### 2.3 Data analyses

To provide a hypothetical framework for population structure and to facilitate discussion of results, sampling sites were assigned to a priori regional groups (*Magellanic*: Straits of Magellan, Falkland Islands, and Burdwood Bank; *Scotia Arc*: Shag Rocks, South Georgia, South Sandwich, Herdman Bank, Discovery Bank, South Orkney; *Antarctic Peninsula*: Elephant Island and Bransfield Strait; *Ross Sea*: Scott A and West Ross Sea; *East Antarctic*: Enderby, Shelf Break, Heard Island Aurora Bank, and Heard Island Coral Bank; Figure 2).

Statistical parsimony networks were calculated in TCS (Clement, Posada, & Crandall, 2000) for each genetic marker with a 95% connection limit and gaps treated as missing data. Likelihood model calculations were performed for COI data in jModelTest (Posada, 2008) with five substitution schemes. The best fitting model was chosen using the Akaike Information Criterion implemented in jModelTest (Guindon & Gascuel, 2003). Both uncorrected and model‐corrected genetic distances between COI haplotypes were calculated in PAUP* (Swofford, 2002).

In order to test for genetic structure in the COI dataset (Hypothesis 1), an Analysis of Molecular Variance (AMOVA) was performed in Arlequin 3.5 (Excoffier & Lischer, 2010) without secondary group assignments. Significance was assessed with 1,000 permutations.

A Mantel test was performed on the COI data in Arlequin 3.5 (Excoffier & Lischer, 2010) to test for isolation‐by‐distance via correlation between Slatkin's linearized Φ_ST_ values and pairwise linear geographic distances calculated from central summary coordinates for each site (Appendix S1). A second Mantel test was performed on samples from the Magellan, Scotia Arc, and Antarctic Peninsula regions to test the correlation between Slatkin's linearized Φ_ST_ and the number of steps between sites. Steps were coded bidirectionally, with one step between each sampling site from north to south in the following order: Straits of Magellan east to South Georgia, south to South Sandwich, then west to Bransfield Strait (Figure 2).

To assess the spatial distribution of genetic clusters (Hypothesis 2) and to test the Antarctic Convergence as a dispersal barrier (Hypothesis 3), a Spatial Analysis of Molecular Variance analysis (SAMOVA) of COI data was performed in SAMOVA v.2 (Dupanloup, Schneider, & Excoffier, 2002). Each putative number of populations (*K*) between 2 and 7 were analyzed, each with 100 repetitions and 10,000 iterations for the simulated annealing process and repeated to evaluate consistency. To identify the appropriate grouping scheme (*K*‐value) for these data, the pseudo‐*F* criterion (Caliński & Harabasz, 1974), and the relative differences in Φ_CT_ were calculated for each *K*.

## 3 RESULTS

**Figure 3:**
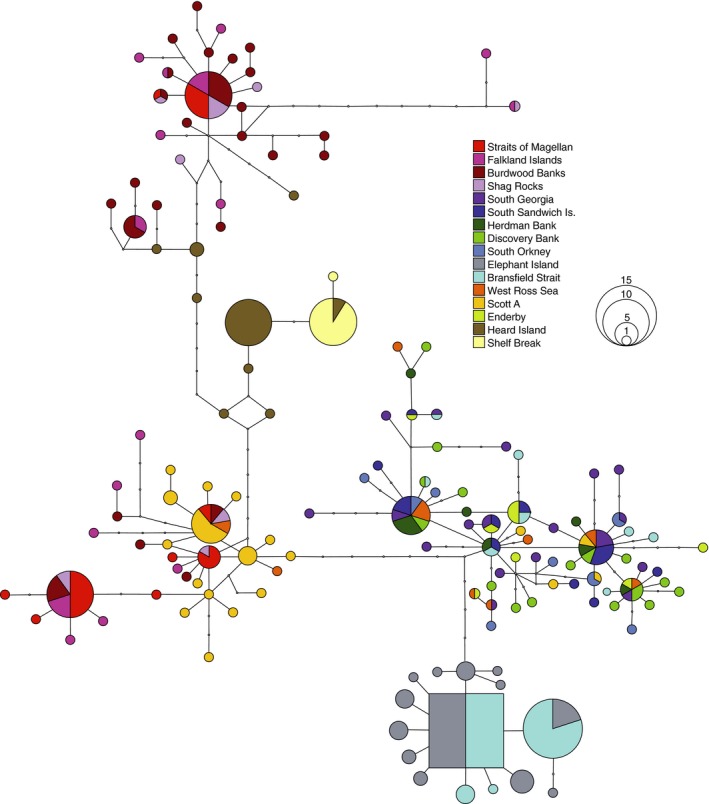
Haplotype network of the COI gene in *Glabraster antarctica*, calculated in TCS. Haplotypes are indicated by colored circles and their frequency is indicated by the size of the circles. Multiple colors indicate haplotypes shared by more than one sampling locality, with sections scaled by frequency. Open circles indicate missing or extinct intermediate haplotypes. The square haplotype indicates the putative ancestral haplotype

**Figure 4:**
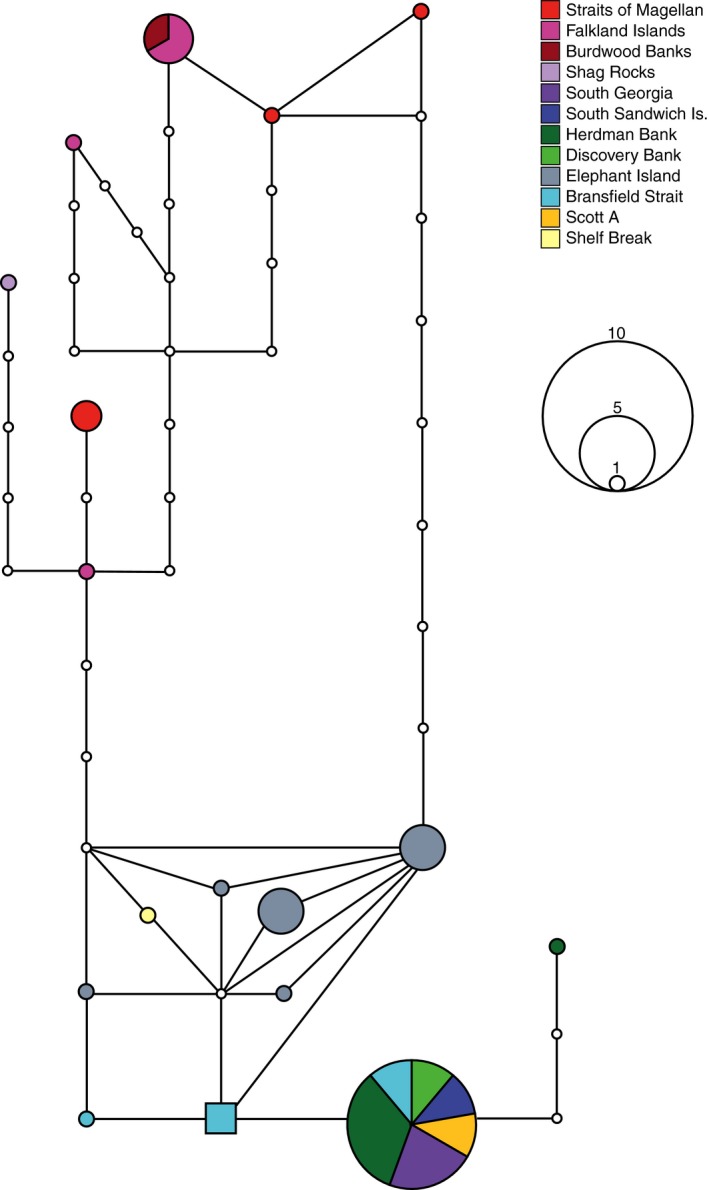
Haplotype network of the ITS2 genetic marker in *Glabraster antarctica*, calculated in TCS. Haplotypes are indicated by colored circles, and their frequency is indicated by the size of the circles. Multiple colors indicate haplotypes shared by more than one sampling locality, with sections scaled by frequency. Open circles indicate missing or extinct intermediate haplotypes. The square haplotype indicates the putative ancestral haplotype

Genetic diversity in COI was extremely high, with 142 haplotypes recovered from 285 individuals. One hundred and eighteen of these haplotypes were private (found in one sampling site), and 106 were singletons (Figure 3). Uncorrected COI genetic distances ranged from 0.15% to 3.36%, and the GTR + I + G AIC best‐fit model‐corrected distances ranged from 0.15% to 3.73%. ITS2 sequencing (*n* = 33) recovered 17 haplotypes, of which 13 were private and 11 singletons (Figure 4). Population statistics are given for each sampling site in Table 1.

**Table 1:** Population statistics of sampled *Glabraster antarctica* calculated for COI data in Arlequin

Group	Site	*n*	Haplotypes	Private haplotypes	Haplotypic diversity (*H)*	Nucleotide diversity (Π)
Magellanic	Straits of Magellan	20	9	4	0.8632	0.01075
	Burdwood Bank	30	25	19	0.9816	0.01221
	Falkland Islands	18	16	11	0.9869	0.01871
	Shag Rocks	9	8	2	0.9722	0.01476
Scotia Arc	South Georgia	19	18	11	0.9942	0.00723
	S. Sandwich Is.	13	10	4	0.9487	0.00491
	Herdman Bank	9	7	3	0.9167	0.00399
	Discovery Bank	17	16	12	0.9926	0.00707
	South Orkney Is.	10	8	5	0.9556	0.00651
Antarctic Peninsula	Elephant Island 1	13	6	5	0.8590	0.00305
	Elephant Island 2	19	8	6	0.8070	0.00195
	Bransfield Strait 1	13	9	3	0.9103	0.00830
	Bransfield Strait 2	19	5	3	0.6842	0.00418
East Antarctic	Heard Is. ‐ Coral Bank	6	6	5	1.0000	0.00947
	Heard Is. ‐ Aurora Bank	13	5	3	0.5385	0.00705
	Shelf Break	11	2	1	0.1818	0.00028
	Enderby	8	7	2	0.9643	0.00671
Ross Sea	Scott A	28	19	16	0.9524	0.00742
	West Ross Sea	10	9	3	0.9778	0.00997

Although morphological variation in size and the presence of abactinal spines exists among the sampled areas (Figure 1), these variants do not correspond to distinct genetic entities and *G. antarctica* appears to constitute a single morphologically variable species.

COI data formed a single, diffuse haplotype network, with some regional clustering of haplotypes (Figure 3). There were some unexpected patterns of structure and connectivity, with several instances of shared haplotypes across geographically distant sites (Figure 3). Surprisingly, Shag Rocks, located south of the Antarctic Convergence, shared haplotypes exclusively with Magellanic sites and not with geographically proximate South Georgia (Figure 3).

The ITS2 network mirrored the pattern in the COI data, forming a single, diffuse network (Figure 4). A common haplotype was shared across Scotia Arc sites (excluding Shag Rocks), the Ross Sea, and the Bransfield Strait, as in the COI network. Burdwood Bank and Falkland Islands samples shared haplotypes, but the Straits of Magellan samples were distributed in diffuse private haplotypes. Other Antarctic Peninsula sites and the East Antarctic site Shelf Break had closely related private haplotypes, separated from the most frequent Scotia Arc haplotype by 1–3 steps.

**Figure 5:**
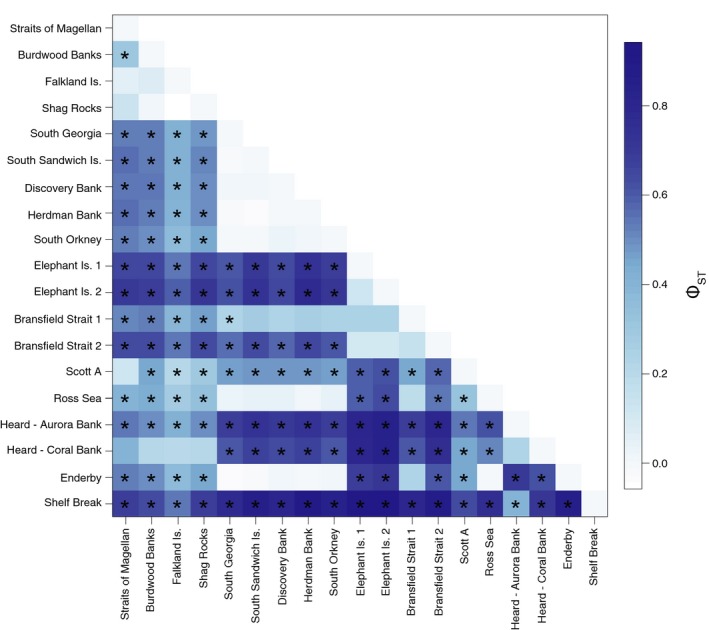
Heat map showing pairwise Φ_ST_ comparisons across all sampled sites of *Glabraster antarctica* in a test of circumpolar panmixia calculated in Arlequin. Darker colors indicate higher pairwise Φ_ST_ values, and asterisks indicate Bonferroni‐corrected significance (*p* < 0.0003)

AMOVA tests of circumpolarity using COI data showed strong differentiation between sites overall (Φ_ST_ = 0.52316, *p* < 0.000, Table 2). Pairwise Φ_ST_ comparisons show a strong affinity between Magellanic sites north of the Antarctic Convergence and Shag Rocks, and instances of affinity between distant sites (Figure 5). Pairwise Φ_ST_ comparisons indicated that much of the within‐region variance (Φ_SC_) was driven by the affinity of Shag Rocks with the Magellanic sites (Figure 5). When Shag Rocks was instead treated as part of the Magellanic region, AMOVA showed a reduction in variance within regions (Φ_SC_ = 0.225; *p* < 0.000) and a corresponding increase in genetic differentiation between regions (Φ_CT_ = 0.428, *p* < 0.000).

**Table 2:** Analysis of molecular variance (AMOVA) testing circumpolar panmixia in COI data for *Glabraster antarctica*

Source of variation	Degrees of freedom	Sum of squares	Variance components	Percentage of variation
Among populations	18	857.511	3.02232 *V*_a_	52.32
Within populations	266	732.770	2.75477 *V*_b_	47.68
Total	284	1590.281	5.77710	
Fixation index (Φ_ST_)		0.52316		
Significance test *V*_a_ & Φ_ST_; 1,023 permutations		*p* < 0.000 ± 0.000		

The Mantel test of isolation‐by‐distance (IBD) indicated low correlation (*r* = 0.262, *p* = 0.02) between pairwise geographic distances and Slatkin's linearized Φ_ST_. A general isolation‐by‐distance model did not adequately explain the variation in pairwise Φ_ST_ among all samples, however, a linear regression of distance versus Slatkin's linearized Φ_ST_ shows that this correlation is stronger when comparisons are made within geographic regions (*R* ^2^ = 0.476), but not when made across regions (*R* ^2^ = 0.025). Thus, IBD is important to explaining genetic connectivity at relatively small spatial scales (i.e., within regions) but other factors influence connectivity over longer distances.

The Mantel test of stepping‐stone dispersal along the Scotia Arc showed strong correlation between step distance and Slatkin's linearized Φ_ST_ (*R* ^2^ = 0.441, *p* = 0.002), which supports the hypothesis that connectivity is maintained across the Scotia Arc via “stepping‐stone” dispersal across shelf habitats. However, the strong genetic structure among the Magellanic, Scotia Arc, and Antarctic Peninsula regions in the AMOVA and SAMOVA analyses indicate that dispersal is limited at broader scales, particularly in the area of the Antarctic Convergence, and overall, we reject Hypothesis 3.

**Figure 6:**
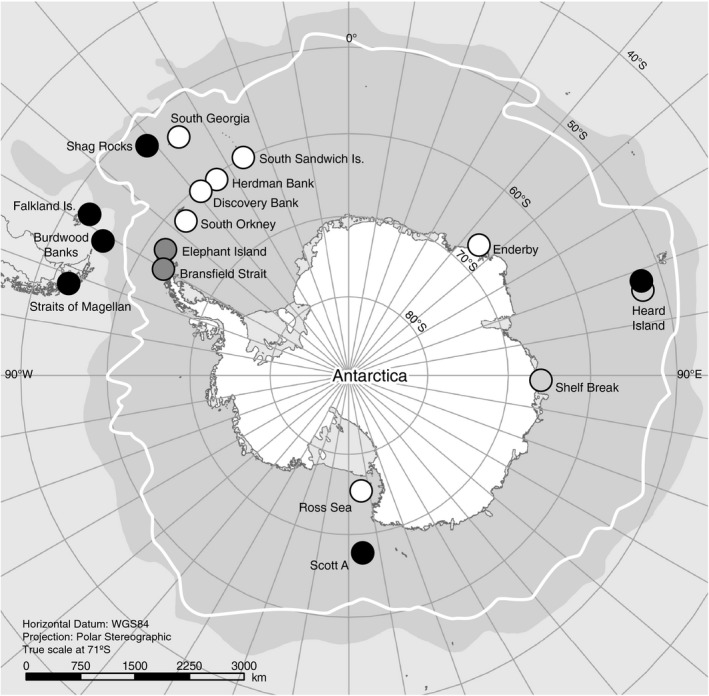
Geographic distribution of SAMOVA groupings for COI data at *k* = 4. Circle shading indicate groupings

SAMOVA results show strong population structure (Φ_CT_) for each *K* from 2 to 7 (*p* < 0.005 in all cases), and the population groupings for each *K* are given in Table 3. The pseudo‐F criterion value is maximized at *K* = 5, however, the rate of change between Φ_CT_ values is maximized at *K* = 4 (Figure 6). At both *K* = 4 and *K* = 5, geographically distant sites are grouped together. Most notably, Magellanic sites including Shag Rocks are grouped with one Heard Island site and Scott A (Ross Sea), and the Scotia Arc group includes West Ross Sea and Enderby (East Antarctica). These results support the hypothesis of circumpolar connectivity despite overall strong population structure. The inclusion of Shag Rocks and two other sites south of the Antarctic Convergence within the Magellanic group indicates that the Antarctic Convergence is an incomplete barrier to gene flow in this species.

**Table 3:** Spatial Analysis of Molecular Variance (SAMOVA) testing subpopulation groupings. A priori regional group assignments are given in italics, individual sampling sites given in regular face

*k*	*F*_CT_	*F*_SC_	pseudo‐*F*	Δ *F*_CT_	Group assignments
2	0.363	0.496	4.014	–	[*Magellan*,* Scotia*,* Peninsula*,* Ross*, Enderby, Heard Coral Bank][Shelf Break, Heard Aurora Bank]
3	0.425	0.334	11.738	0.062	[*Magellan*, Shag Rocks, Scott A, Heard Coral Bank][*Scotia*,* Peninsula*, West Ross, Enderby][Shelf Break, Heard Aurora Bank]
4	0.497	0.200	18.605	0.071	[*Magellan*, Shag Rocks, Scott A, Heard Coral Bank][*Scotia*, West Ross, Enderby][*Peninsula*][Shelf Break, Heard Aurora Bank]
5	0.540	0.093	30.390	0.043	[Straits of Magellan, Scott A][Falklands, Burdwood Bank, Shag Rocks, Heard Coral Bank][*Scotia*, West Ross, Enderby][*Peninsula*][Shelf Break, Heard Aurora Bank]
6	0.550	0.069	29.456	0.010	[Straits of Magellan, Scott A][Falklands, Burdwood Bank, Shag Rocks][*Scotia*, West Ross, Enderby][*Peninsula*][*Heard Island*][Shelf Break]
7	0.553	0.061	26.202	0.004	[Straits of Magellan, Scott A][Falklands, Burdwood Bank, Shag Rocks][*Scotia*, West Ross, Enderby][*Peninsula*][Heard Aurora Bank][Heard Coral Bank][Shelf Break]

## 4 DISCUSSION

### 4.1 A single circum‐Antarctic species

Morphological variants of *G. antarctica* do not reflect distinct genetic entities that would support the presence of cryptic species. The Antarctic Peninsula morphotype (Figure 1a) is small, with strong abactinal spination, and shares haplotypes with the large Scotia Arc samples lacking abactinal spines (Figures 2, 3, 4), the latter corresponding to the synonymized subspecies *G. antarctica glabra* (Sladen, 1889). Magellanic specimens are bright red‐orange with distinct abactinal spination and correspond to the synonymized subspecies *G. antarctica magellanica* (Studer 1876; Figure 1c,d). Furthermore, COI distances among all samples are less than 4%, and prior studies on echinoderm “barcode gaps” have shown interspecific distances of at least 5.6%, with a mean of 10.9% (Hebert, Ratnasingham, & de Waard, 2003; Meier, Zhang, Ali, & Zamudio, 2008). Our results support the synonymy of these subspecies (Mah & Foltz, 2014), and the view that *G. antarctica* represents a single, morphologically variable, circum‐Antarctic species.

*Glabraster antarctica* is characterized by high genetic diversity, many private haplotypes, and substantial population structure; yet, there are low genetic distances among haplotypes, and subpopulations are distributed across broad spatial scales. Although we reject the hypothesis of panmixia in this species (Hypothesis 1), these results demonstrate enough intermittent genetic connectivity to maintain a structured circumpolar species.

### 4.2 Structure in circumpolarity

The larvae of *G. antarctica* are highly buoyant, with a PLD of 60 days (Bosch, 1989), allowing dispersal in the fastest upper portion of the Antarctic Circumpolar Current (Ivchenko & Richards, 1996) and potentially driving the long‐distance connectivity patterns seen in part of the haplotype network. The dispersal potential over a single generation in *G. antarctica* is unknown, but larval behavior is important to realized dispersal ability in marine species (reviewed by Levin, 2006), and complexities in the population structure of this species indicate multiple factors at play. SAMOVA results indicate genetic connectivity among extremely distant sampling sites, and several geographically proximate sites were grouped separately in the SAMOVA analysis with significant pairwise Φ_ST_ in the AMOVA analysis. In East Antarctica, Shelf Break is genetically isolated from another continental site Enderby (Figure 5) and is instead grouped with the Aurora Bank Heard Island site in the SAMOVA analysis (Table 3). This pattern may be driven by entrainment of Shelf Break larvae in the Prydz Bay gyre (Heywood, Sparrow, Brown, & Dickson, 1999; Nicol, Pauly, Bindoff, & Strutton, 2000). Our Enderby site is well outside the western limit of this gyre (45°E, Figure 2) and is grouped with Scotia Arc sites in the SAMOVA analysis, while the Shelf Break site is located within the region influenced by the Prydz Bay gyre. A Prydz Bay‐Scotia Arc connection was noted in the octopus *Pareledone turqueti* (Strugnell et al., 2012), and a similar pattern of long‐distance connectivity between East Antarctica and the Antarctic Peninsula was found in the amphipod *Eusirus giganteus*, contrasting with strong genetic structure within East Antarctica in *Eusirus perdentatus* (Baird, Miller, & Stark, 2011).

Antarctic Peninsula sites are incompletely isolated from Scotia Arc sites, and this may reflect competing influences of the ACC and Antarctic coastal countercurrent through Bransfield Strait. Countercurrent measurements have shown strong westward flow of surface waters through the Bransfield Strait and past Elephant Island (von Gyldenfeldt, Fahrbach, García, & Schröder, 2002), which may drive larval retention within the Antarctic Peninsula area and limit dispersal to the Scotia Arc.

Long‐distance connectivity among distant sites suggests that the ACC and coastal countercurrent may drive circumpolar larval transport and maintain genetic connection between distant regions. Kelp‐rafting peracarid crustaceans have shown a similar pattern of circumpolar dispersal via ACC transport (Nikula et al., 2010). Corroboration of COI results with further nuclear data is necessary to verify patterns of connectivity in *G. antarctica*.

### 4.3 The “stepping‐stones” of the Scotia Arc

Stepwise dispersal across the shelf habitats of the Scotia Arc was strongly correlated with Slatkin's linearized Φ_ST_ in the Mantel test, which supports the stepping‐stone hypothesis: that connectivity across the Antarctic Convergence may be facilitated by the shallow shelf habitat available along the Scotia Arc. However, the strong genetic isolation of the Magellanic region, Scotia Arc, and Antarctic Peninsula in pairwise Φ_ST_ comparisons indicates that other forces, such as currents, fronts, and gyres, may have a stronger influence on genetic connectivity across the Scotia Arc than habitat availability. Long‐distance connectivity between both the Magellanic region and the Scotia Arc region with distant Ross Sea and East Antarctic regions also supports this view.

Connectivity patterns in *G. antarctica* indicate that the Magellanic zoogeographic province may include Shag Rocks, despite its location south of the Antarctic Convergence and proximity to South Georgia. Tests of population differentiation across the Antarctic Convergence show a strong genetic break between Shag Rocks and South Georgia for *G. antarctica* (Table 3, Figure 5); Magellanic haplotypes are shared with Shag Rocks but no other Scotia Arc samples (Figures 3 and 4).Divergence between Shag Rocks and South Georgia was also recovered for the octopus *Pareldone turqueti* (Allcock, Brierley, Thorpe, & Rodhouse, 1997; Strugnell, Allcock, & Watts, 2017), and these authors initially proposed the deep water (maximum 1750 m) separating the sites as a strong barrier, limiting pelagic dispersal. However, other organisms with long pelagic stages have shown connectivity across this putative barrier, for example fish species *Dissostichus eleginoides* (Shaw, Arkhipkin, & Al‐Khairulla, 2004) and *Champsocephalus gunnari* (Kuhn & Gaffney, 2006), and the nemertean *Parborlasia corrugatus*, which has a long PLD and extended pelagicism after metamorphosis (Thornhill, Mahon, Norenburg, & Halanych, 2008).

The unique current processes at play in the vicinity of South Georgia may explain the genetic isolation of Shag Rocks from South Georgia in *G. antarctica*. Currents immediately between Shag Rocks and South Georgia (~38°W) of Antarctic Intermediate Water show a relatively strong southward flow of 20 cm/s (Arhan, Naveira Garabato, Heywood, & Stevens, 2002). Furthermore, the ACC deflects northward on the east side of South Georgia before returning to eastward flow (Orsi, Whitworth, & Nowlin, 1995; Thorpe, Heywood, Brandon, & Stevens, 2002), and the combination of these factors may entrain short‐lived larvae from South Georgia and contribute to its isolation from Shag Rocks despite geographic proximity.

Shag Rocks is situated in an area with relatively little seasonal and annual variation in the location of the Antarctic Convergence (Moore, Abbott, & Richman, 1999), and it is therefore unlikely that seasonality contributes to the connectivity of Shag Rocks with the Magellan region. Mesoscale eddies have been suggested as a transport mechanism for marine plankton across the Antarctic Convergence (Barnes, Hodgson, Convey, Allen, & Clarke, 2006), however, these are rare events and may not adequately explain the strong affinity of Shag Rocks with northern sites. Further work is needed to clarify the mechanisms by which *G. antarctica* populations are maintained across this strong dispersal barrier.

The broader Magellanic region concept suggested by our results contrasts with evidence of genetic isolation between the Straits of Magellan and the Falkland Islands in the brooding isopod *Serolis paradoxa* (Leese, Kop, Wägele, & Held, 2008). Shag Rocks is both physically distant from other Magellanic sites and thermally separated by the Antarctic Convergence (Moore et al., 1999; Smith, Stevens, Heywood, & Meredith, 2010), and the Subantarctic Front to the east of Burdwood Bank (Smith et al., 2010), challenging the idea that pelagic dispersal is limited across these frontal zones.

Overall, our study demonstrates that localized current regimes and water mass isolation may drive fine‐scale regional genetic isolation, for example, of the Scotia Arc region from the Magellanic region, and affect even those species with planktonic development and broad depth distributions. Several instances of connectivity among geographically distant regions demonstrate the dispersive influence of the ACC on planktonic developers. The striking diversity, genetic structure, and complex pattern of circumpolarity in *G. antarctica* is driven by the unique oceanographic and historical features of Antarctica and these complexities should be carefully considered in conservation planning.

## DATA ACCESSIBILITY

DNA sequences: NCBI Genbank Accession numbers KP663829‐KP664113 (COI) and KT459301‐KT459333 (ITS2). Additional specimen, collections, and locality data are given in Appendix S1.
