# Supplementary material for: The Antarctic Circumpolar Current isolates and connects: Structured circumpolarity in the sea star Glabraster antarctica
Source: Ecol Evol. 2018 Oct 12;8(21):10621–33. doi: 10.1002/ece3.4551 (PMC6238125; doi:10.1002/ece3.4551)
Supplement: Supplementary file 1 [file ECE3-8-10621-s001.doc]

Moore, J. M., J. I. Carvajal, G. W. Rouse, & N. G. Wilson. The Antarctic Circumpolar Current isolates and connects: structured circumpolarity in the sea star *Glabraster antarctica*. Ecology & Evolution.

Appendix S1. Specimen depth, locality, voucher deposition and GenBank accession numbers for all sampled individuals. SIO BIC = Scripps Institution of Oceanography Benthic Invertebrates Collection, La Jolla, California, USA. NIWA = National Institute of Water & Atmosphere, Wellington, New Zealand.

| Group | Locality | Station | Depth (m) | Geographic coordinates | Field  Number | Voucher Catalog  Number | COI  GenBank ID | ITS2  GenBank ID |
| --- | --- | --- | --- | --- | --- | --- | --- | --- |
| Magellanic | Burdwood Bank | BB1 | 156 | -54.683090°, -60.932607° | S5576 |  | KP663983 |  |
|  |  |  | 156 |  | S5577 |  | KP663984 |  |
|  |  |  | 156 |  | S5578 | SIO BIC E5323 | KP663985 |  |
|  |  |  | 156 |  | S5579 |  | KP663986 |  |
|  |  |  | 156 |  | S5580 |  | KP663987 |  |
|  |  |  | 156 |  | S5582 |  | KP663988 |  |
|  |  |  | 156 |  | S5583 |  | KP663989 |  |
|  |  |  | 156 |  | S5584 |  | KP663990 |  |
|  |  |  | 156 |  | S5585 |  | KP663991 |  |
|  |  |  | 156 |  | S5586 |  | KP663992 |  |
|  |  |  | 156 |  | S5636 |  | KP663993 |  |
|  |  |  | 156 |  | S5637 |  | KP663994 |  |
|  |  |  | 156 |  | S5638 |  | KP663995 |  |
|  |  |  | 156 |  | S5639 |  | KP663996 |  |
|  |  |  | 156 |  | S5640 |  | KP663997 |  |
|  |  |  | 156 |  | S5641 |  | KP663998 |  |
|  |  |  | 156 |  | S5642 |  | KP663999 |  |
|  |  |  | 156 |  | S5643 |  | KP664000 | KT459307 |
|  |  |  | 156 |  | S5644 |  | KP664001 |  |
|  |  |  | 156 |  | S5645 |  | KP664002 |  |
|  |  |  | 156 |  | S5646 |  | KP664003 |  |
|  |  |  | 156 |  | S5647 |  | KP664004 |  |
|  |  |  | 156 |  | S5648 | SIO BIC E5324 | KP664005 |  |
|  |  |  | 156 |  | S5649 |  | KP664006 |  |
|  |  |  | 156 |  | S5650 |  | KP664007 |  |
|  |  |  | 156 |  | S5651 |  | KP664008 |  |
|  |  |  | 156 |  | S5652 |  | KP664009 |  |
|  |  |  | 156 |  | S5653 |  | KP664010 |  |
|  |  |  | 156 |  | S5654 |  | KP664011 |  |
|  |  |  | 156 |  | S5655 | SIO BIC E5327 | KP664012 |  |
|  | Falkland Islands | FIE | 124 | -51.683700°, -57.477517° | S20839 | SIO BIC E6413 | KP664096 |  |
|  |  |  | 124 |  | S20840 | SIO BIC E5769 | KP664097 |  |
|  |  |  | 138 |  | S20871 |  | KP664098 | KT459305 |
|  |  |  | 138 |  | S20872 |  | KP664099 | KT459306 |
|  |  |  | 138 |  | S20873 |  | KP664100 | KT459310 |
|  |  |  | 138 |  | S20874 |  | KP664101 |  |
|  |  |  | 140 |  | S20925 |  | KP664102 | KT459304 |
|  |  |  | 140 |  | S20926 |  | KP664103 |  |
|  |  |  | 140 |  | S20927 |  | KP664104 |  |
|  |  |  | 140 |  | S20929 |  | KP664105 |  |
|  |  |  | 140 |  | S20930 |  | KP664106 |  |
|  |  |  | 140 |  | S20931 |  | KP664107 |  |
|  |  |  | 140 |  | S20932 | SIO BIC E6524 | KP664108 |  |
|  |  |  | 140 |  | S20933 |  | KP664109 |  |
|  |  |  | 140 |  | S20934 |  | KP664110 |  |
|  |  |  | 141 |  | S20939 | SIO BIC E6525 | KP664111 |  |
|  |  |  | 141 |  | S20940 | SIO BIC E6406 | KP664112 |  |
|  |  |  | 134 |  | S20978 |  | KP664113 |  |
|  | Straits of Magellan | SM1 | 285 | -53.557306°, -70.305839° | S20019 | SIO BIC E6493 | KP664067 |  |
|  |  |  | 285 |  | S20020 | SIO BIC E6487 | KP664068 |  |
|  |  |  | 285 |  | S20021 | SIO BIC E6467 | KP664069 |  |
|  |  |  | 285 |  | S20024 | SIO BIC E6416 | KP664070 |  |
|  |  |  | 270 |  | S20071 | SIO BIC E6471 | KP664071 |  |
|  |  |  | 270 |  | S20072 | SIO BIC E6470 | KP664072 |  |
|  |  |  | 270 |  | S20073 | SIO BIC E6469 | KP664073 |  |
|  |  |  | 270 |  | S20076 | SIO BIC E6489 | KP664074 |  |
|  |  |  | 270 |  | S20077 | SIO BIC E6492 | KP664075 | KT459308 |
|  |  |  | 270 |  | S20078 | SIO BIC E6490 | KP664076 |  |
|  |  |  | 75 |  | S20141 | SIO BIC E6453 | KP664077 |  |
|  |  |  | 75 |  | S20142 | SIO BIC E6491 | KP664078 |  |
|  |  |  | 75 |  | S20143 | SIO BIC E6456 | KP664079 |  |
|  |  |  | 75 |  | S20144 | SIO BIC E6455 | KP664080 | KT459301 |
|  |  |  | 75 |  | S20145 | SIO BIC E6403 | KP664081 | KT459302 |
|  |  |  | 75 |  | S20146 | SIO BIC E6449 | KP664082 |  |
|  |  |  | 75 |  | S20147 | SIO BIC E6454 | KP664083 | KT459309 |
|  |  |  | 75 |  | S20148 | SIO BIC E6452 | KP664084 |  |
|  |  |  | 75 |  | S20149 | SIO BIC E6450 | KP664085 |  |
|  |  |  | 75 |  | S20150 | SIO BIC E6451 | KP664086 |  |
|  | Shag Rocks | SR4 | 129 | -53.560867°, -41.654250° | S20364 |  | KP664087 |  |
|  |  |  | 129 |  | S20365 | SIO BIC E6446 | KP664088 |  |
|  |  |  | 125 |  | S20371 |  | KP664089 |  |
|  |  |  | 125 |  | S20372 |  | KP664090 |  |
|  |  |  | 130 |  | S20405 | SIO BIC E6442 | KP664091 |  |
|  |  |  | 129 |  | S20406 | SIO BIC E6526 | KP664092 | KT459303 |
|  |  |  | 127 |  | S20426 |  | KP664093 |  |
|  |  |  | 126 |  | S20430 |  | KP664094 |  |
|  |  |  | 126 |  | S20476 |  | KP664095 |  |
| Scotia Arc | South Georgia | SG2a | 140 | -53.785345°, -37.215072° | S3284 |  | KP663935 |  |
|  |  |  | 140 |  | S3285 |  | KP663936 |  |
|  |  |  | 140 |  | S3286 |  | KP663937 |  |
|  |  |  | 140 |  | S3287 |  | KP663938 |  |
|  |  |  | 140 |  | S3289 |  | KP663939 | KT459326 |
|  |  |  | 140 |  | S3291 |  | KP663940 |  |
|  |  |  | 143 |  | S3912 |  | KP663941 |  |
|  |  |  | 143 |  | S3913 | SIO BIC E5322 | KP663942 |  |
|  |  |  | 143 |  | S3914 |  | KP663943 |  |
|  |  |  | 143 |  | S3915 |  | KP663944 |  |
|  |  |  | 143 |  | S3916 |  | KP663945 |  |
|  |  |  | 143 |  | S3919 |  | KP663946 |  |
|  |  |  | 143 |  | S3921 |  | KP663947 |  |
|  |  |  | 143 |  | S3923 |  | KP663948 |  |
|  |  |  | 143 |  | S3924 |  | KP663949 |  |
|  |  |  | 143 |  | S3925 |  | KP663950 |  |
|  |  |  | 143 |  | S3926 |  | KP663951 |  |
|  |  |  | 143 |  | S3928 | SIO BIC E5321 | KP663952 | KT459327 |
|  |  |  | 143 |  | S3929 |  | KP663953 |  |
|  | South Sandwich | SS2 | 161 | -58.419747°, -26.244962° | S0168 |  | KP663915 |  |
|  |  |  | 161 |  | S0169 |  | KP663916 | KT459324 |
|  |  |  | 161 |  | S0170 |  | KP663917 |  |
|  |  |  | 161 |  | S0171 |  | KP663918 |  |
|  |  |  | 161 |  | S0476 | SIO BIC E5320 | KP663919 |  |
|  |  |  | 161 |  | S0477 |  | KP663920 |  |
|  |  |  | 161 |  | S0478 |  | KP663921 |  |
|  |  |  | 161 |  | S0519 |  | KP663922 |  |
|  |  |  | 161 |  | S0520 |  | KP663923 |  |
|  |  |  | 161 |  | S0335 |  | KP663924 |  |
|  |  |  | 161 |  | S0336 |  | KP663925 |  |
|  |  |  | 161 |  | S0597 |  | KP663926 |  |
|  |  |  | 161 |  | S0798 |  | KP663927 |  |
|  | Herdman Bank | HB1 | 520 | -59.881793°, -32.424997° | S4401 |  | KP663970 |  |
|  |  |  | 520 |  | S4402 |  | KP663971 |  |
|  |  |  | 520 |  | S4403 |  | KP663972 |  |
|  |  |  | 520 |  | S4404 |  | KP663973 | KT459328 |
|  |  |  | 553 |  | S4421 |  | KP663974 |  |
|  |  |  | 553 |  | S4425 |  | KP663975 | KT459311 |
|  |  |  | 553 |  | S4426 |  | KP663976 | KT459329 |
|  |  |  | 553 |  | S4428 |  | KP663977 |  |
|  |  |  | 600 |  | S4485 | SIO BIC E5325 | KP663978 | KT459330 |
|  | Discovery Bank | DB1 | 461 | -60.116822°, -34.891323° | S1837 | SIO BIC E5782 | KP663928 |  |
|  |  |  | 461 |  | S1838 | SIO BIC E5783 | KP663929 |  |
|  |  |  | 461 |  | S1839 |  | KP663930 | KT459325 |
|  |  |  | 762 |  | S1852 |  | KP663931 |  |
|  |  |  | 762 |  | S1853 |  | KP663932 |  |
|  |  |  | 379 |  | S1978 |  | KP663933 |  |
|  |  |  | 379 |  | S1980 |  | KP663934 |  |
|  |  |  | 379 |  | S4012 |  | KP663954 |  |
|  |  |  | 452 |  | S4106 |  | KP663955 |  |
|  |  |  | 452 |  | S4107 |  | KP663956 |  |
|  |  |  | 397 |  | S4114 |  | KP663957 |  |
|  |  |  | 397 |  | S4115 |  | KP663958 |  |
|  |  |  | 397 |  | S4116 |  | KP663959 |  |
|  |  |  | 391 |  | S4170 |  | KP663966 |  |
|  |  |  | 439 |  | S4301 |  | KP663967 |  |
|  |  |  | 439 |  | S4302 |  | KP663968 |  |
|  |  |  | 439 |  | S4303 |  | KP663969 |  |
|  | South Orkney | AMLR31-28 | 247-261 | -61.0368°,  -44.7085° | SO3128-A |  | KP663829 |  |
|  |  |  | 247-261 |  | SO3128-B |  | KP663851 |  |
|  |  |  | 247-261 |  | SO3128-E |  | KP663852 |  |
|  |  |  | 247-261 |  | SO3128-F |  | KP663853 |  |
|  |  |  | 247-261 |  | SO3128-G |  | KP663854 |  |
|  |  |  | 247-261 |  | SO3128-H |  | KP663855 |  |
|  |  |  | 247-261 |  | SO3128-I |  | KP663856 |  |
|  |  |  | 247-261 |  | SO3128-J |  | KP663857 |  |
|  |  |  | 247-261 |  | SO3128-K |  | KP663858 |  |
|  |  |  | 247-261 |  | SO3128-M |  | KP663859 |  |
| Antarctic Peninsula | Elephant Islands | EI1 | 223 | -61.183659°, -54.226290° | S6871 | SIO BIC E5337 | KP664054 |  |
|  |  |  | 223 |  | S6872 | SIO BIC E5328 | KP664055 |  |
|  |  |  | 223 |  | S6873 | SIO BIC E5336 | KP664056 |  |
|  |  |  | 223 |  | S6874 | SIO BIC E5329 | KP664057 | KT459314 |
|  |  |  | 223 |  | S6875 | SIO BIC E5338 | KP664058 |  |
|  |  |  | 223 |  | S6876 | SIO BIC E5334 | KP664059 | KT459321 |
|  |  |  | 223 |  | S6877 | SIO BIC E5332 | KP664060 |  |
|  |  |  | 223 |  | S6878 | SIO BIC E5331 | KP664061 |  |
|  |  |  | 223 |  | S6879 | SIO BIC E5333 | KP664062 |  |
|  |  |  | 223 |  | S6880 | SIO BIC E5339 | KP664063 | KT459320 |
|  |  |  | 223 |  | S6881 | SIO BIC E5330 | KP664064 |  |
|  |  |  | 223 |  | S6882 |  | KP664065 |  |
|  |  |  | 223 |  | S6883 | SIO BIC E5335 | KP664066 |  |
|  |  | EI2 | 143 | -61.338772°, -55.624935° | S6601 |  | KP664032 |  |
|  |  |  | 143 |  | S6602 |  | KP664033 | KT459315 |
|  |  |  | 143 |  | S6603 |  | KP664034 |  |
|  |  |  | 143 |  | S6604 |  | KP664035 | KT459318 |
|  |  |  | 143 |  | S6605 |  | KP664036 | KT459316 |
|  |  |  | 143 |  | S6606 |  | KP664037 | KT459319 |
|  |  |  | 143 |  | S6607 |  |  | KT459312 |
|  |  |  | 143 |  | S6608 |  | KP664038 | KT459313 |
|  |  |  | 143 |  | S6609 |  | KP664039 |  |
|  |  |  | 143 |  | S6610 |  | KP664040 |  |
|  |  |  | 143 |  | S6611 |  | KP664041 |  |
|  |  |  | 143 |  | S6612 |  | KP664042 |  |
|  |  |  | 143 |  | S6613 |  | KP664043 |  |
|  |  |  | 143 |  | S6614 |  | KP664044 |  |
|  |  |  | 143 |  | S6615 |  | KP664045 |  |
|  |  |  | 143 |  | S6616 |  | KP664046 |  |
|  |  |  | 143 |  | S6617 |  | KP664047 |  |
|  |  |  | 143 |  | S6618 |  | KP664048 |  |
|  |  |  | 143 |  | S6619 |  | KP664049 |  |
|  |  |  | 143 |  | S6620 |  | KP664050 |  |
|  | Bransfield Strait | BS1 | 292 | -62.806495°, -57.271218° | S4129 |  | KP663960 |  |
|  |  |  | 292 |  | S4130 |  | KP663961 |  |
|  |  |  | 292 |  | S4131 |  | KP663962 |  |
|  |  |  | 292 |  | S4132 |  | KP663963 |  |
|  |  |  | 292 |  | S4133 |  | KP663964 |  |
|  |  |  | 292 |  | S4134 |  | KP663965 |  |
|  |  |  | 163 |  | S4702 |  | KP663979 |  |
|  |  |  | 163 |  | S4703 |  | KP663980 |  |
|  |  |  | 163 |  | S4704 |  | KP663981 |  |
|  |  |  | 163 |  | S4705 |  | KP663982 |  |
|  |  |  | 247 |  | S6686 |  | KP664051 |  |
|  |  |  | 247 |  | S6687 |  | KP664052 |  |
|  |  |  | 247 |  | S6688 |  | KP664053 |  |
|  |  | BS2 | 199 | -63.330119°, -59.876300° | S5956 |  | KP664013 |  |
|  |  |  | 199 |  | S5957 |  |  | KT459332 |
|  |  |  | 199 |  | S5958 |  | KP664014 |  |
|  |  |  | 199 |  | S5959 |  | KP664015 |  |
|  |  |  | 199 |  | S5960 |  | KP664016 | KT459322 |
|  |  |  | 199 |  | S5961 |  | KP664017 |  |
|  |  |  | 199 |  | S5962 |  | KP664018 |  |
|  |  |  | 199 |  | S5964 |  | KP664019 | KT459333 |
|  |  |  | 199 |  | S5965 |  | KP664020 |  |
|  |  |  | 199 |  | S5966 |  | KP664021 |  |
|  |  |  | 199 |  | S5967 |  | KP664022 |  |
|  |  |  | 199 |  | S5968 |  | KP664023 |  |
|  |  |  | 199 |  | S5969 |  | KP664024 |  |
|  |  |  | 199 |  | S5970 |  | KP664025 | KT459331 |
|  |  |  | 213 |  | S6293 |  | KP664026 |  |
|  |  |  | 213 |  | S6294 |  | KP664027 |  |
|  |  |  | 213 |  | S6295 |  | KP664028 |  |
|  |  |  | 213 |  | S6296 |  | KP664029 |  |
|  |  |  | 213 |  | S6298 |  | KP664030 |  |
|  |  |  | 213 |  | S6299 |  | KP664031 |  |
| Ross Sea | Scott A |  | 421 | -67.819106°, -179.629181° | N0013 | NIWA  39175 | KP663880 |  |
|  |  |  | 329-334 |  | N0022 | NIWA  36660 | KP663881 |  |
|  |  |  | 421 |  | N0026 | NIWA  38323 | KP663882 |  |
|  |  |  | 421 |  | N0027 | NIWA  38323 | KP663883 |  |
|  |  |  | 421 |  | N0028 | NIWA  38278 | KP663884 | KT459323 |
|  |  |  | 329-334 |  | N0029 | NIWA  38278 | KP663885 |  |
|  |  |  | 329-334 |  | N0030 | NIWA  85182 | KP663886 |  |
|  |  |  | 402-406 |  | N0031 | NIWA  85182 | KP663887 |  |
|  |  |  | 402-406 |  | N0032 | NIWA  85182 | KP663888 |  |
|  |  |  | 402-406 |  | N0033 | NIWA  85180 | KP663889 |  |
|  |  |  | 358-382 |  | N0044 | NIWA  85181 | KP663890 |  |
|  |  |  | 358-382 |  | N0046 | NIWA  85181 | KP663891 |  |
|  |  |  | 358-382 |  | N0047 | NIWA  85181 | KP663892 |  |
|  |  |  | 358-382 |  | N0048 | NIWA  85179 | KP663893 |  |
|  |  |  | 402-406 |  | N0049 | NIWA  85179 | KP663894 |  |
|  |  |  | 402-406 |  | N0050 | NIWA  85179 | KP663895 |  |
|  |  |  | 402-406 |  | N0051 | NIWA  85179 | KP663896 |  |
|  |  |  | 402-406 |  | N0053 | NIWA  85179 | KP663897 |  |
|  |  |  | 402-406 |  | N0054 | NIWA  85179 | KP663880 |  |
|  |  |  | 402-406 |  | N0055 | NIWA  85179 | KP663881 |  |
|  |  |  | 402-406 |  | N0056 | NIWA  85179 | KP663882 |  |
|  |  |  | 402-406 |  | N0057 | NIWA  85179 | KP663883 |  |
|  |  |  | 402-406 |  | N0059 | NIWA  85179 | KP663884 |  |
|  |  |  | 402-406 |  | N0060 | NIWA  85179 | KP663885 |  |
|  |  |  | 402-406 |  | N0061 | NIWA  85179 | KP663886 |  |
|  |  |  | 402-406 |  | N0062 | NIWA  85179 | KP663887 |  |
|  |  |  | 402-406 |  | N0063 | NIWA  85179 | KP663888 |  |
|  |  |  | 402-406 |  | N0064 | NIWA  85179 | KP663889 |  |
|  | West Ross Sea |  | 360 | -76.598478°, 176.815560° | N0025 | NIWA  38312 | KP663863 |  |
|  |  |  | 360 |  | N0023 | NIWA  36660 | KP663862 |  |
|  |  |  | 365-369 |  | N0035 | NIWA  85180 | KP663872 |  |
|  |  |  | 365-369 |  | N0036 | NIWA  85180 | KP663873 |  |
|  |  |  | 365-369 |  | N0038 | NIWA  85180 | KP663874 |  |
|  |  |  | 365-369 |  | N0039 | NIWA  85180 | KP663875 |  |
|  |  |  | 365-369 |  | N0040 | NIWA  85180 | KP663876 |  |
|  |  |  | 365-369 |  | N0041 | NIWA  85180 | KP663877 |  |
|  |  |  | 365-369 |  | N0042 | NIWA  85180 | KP663878 |  |
|  |  |  | 365-369 |  | N0043 | NIWA  85181 | KP663879 |  |
| East Antarctica | Heard Island Aurora Bank |  | 127 | -52.482993°, 72.040523° | AAJ080 |  | KP663838 |  |
|  |  |  | 127 |  | AAJ083 |  | KP663839 |  |
|  |  |  | 127 |  | AAJ084 |  | KP663840 |  |
|  |  |  | 127 |  | AAJ085 |  | KP663841 |  |
|  |  |  | 127 |  | AAJ086 |  | KP663842 |  |
|  |  |  | 127 |  | AAJ087 |  | KP663843 |  |
|  |  |  | 127 |  | AAJ088 |  | KP663844 |  |
|  |  |  | 127 |  | AAJ089 |  | KP663845 |  |
|  |  |  | 127 |  | AAJ090 |  | KP663846 |  |
|  |  |  | 127 |  | AAJ091 |  | KP663847 |  |
|  |  |  | 127 |  | AAJ095 |  | KP663848 |  |
|  |  |  | 127 |  | AAJ096 |  | KP663849 |  |
|  |  |  | 222 |  | PNG556 |  | KP663909 |  |
|  | Heard Island Coral Bank |  | 160 | -51.938333°, 71.286167° | AAJ097 |  | KP663850 |  |
|  |  |  | 296 |  | PNG557 |  | KP663910 |  |
|  |  |  | 293 |  | PNG558 |  | KP663911 |  |
|  |  |  | 293 |  | PNG559 |  | KP663912 |  |
|  |  |  | 293 |  | PNG560 |  | KP663913 |  |
|  |  |  | 293 |  | PNG561 |  | KP663914 |  |
|  | Enderby |  | 247 | -67.212500°, 44.622121° | AA116 |  | KP663830 |  |
|  |  |  | 223 |  | AA118 |  | KP663831 |  |
|  |  |  | 223 |  | AA119 |  | KP663832 |  |
|  |  |  | 223 |  | AA120 |  | KP663833 |  |
|  |  |  | 223 |  | AA121 |  | KP663834 |  |
|  |  |  | 260 |  | AA126 |  | KP663835 |  |
|  |  |  | 260 |  | AA127 |  | KP663836 |  |
|  |  |  | 260 |  | AA128 |  | KP663837 |  |
|  | Shelf Break |  | 522.4 | -65.835059°, 89.542375° | PNG532 |  | KP663898 |  |
|  |  |  | 522.4 |  | PNG533 |  | KP663899 |  |
|  |  |  | 522.4 |  | PNG534 |  | KP663900 |  |
|  |  |  | 403.6 |  | PNG538 |  | KP663901 |  |
|  |  |  | 403.6 |  | PNG539 |  | KP663902 |  |
|  |  |  | 727.6 |  | PNG540 |  | KP663903 |  |
|  |  |  | 727.6 |  | PNG544 |  | KP663904 |  |
|  |  |  | 526.8 |  | PNG545 |  |  | KT459317 |
|  |  |  | 526.8 |  | PNG546 |  | KP663905 |  |
|  |  |  | 526.8 |  | PNG548 |  | KP663906 |  |
|  |  |  | 526.8 |  | PNG550 |  | KP663907 |  |
|  |  |  | 526.8 |  | PNG551 |  | KP663908 |  |
